# Ambient Healthcare Approach with Hybrid Whale Optimization Algorithm and Naïve Bayes Classifier

**DOI:** 10.3390/s21134579

**Published:** 2021-07-04

**Authors:** Majed Alwateer, Abdulqader M. Almars, Kareem N. Areed, Mostafa A. Elhosseini, Amira Y. Haikal, Mahmoud Badawy

**Affiliations:** 1 College of Computer Science and Engineering, Taibah University, Yanbu 46421, Saudi Arabia; MWATEER@taibahu.edu.sa (M.A.); Amars@taibahu.edu.sa (A.M.A.); melhosseini@mans.edu.eg (M.A.E.); 2 Computers and Control Systems Engineering Department, Faculty of Engineering, Mansoura University, Mansoura 35516, Egypt; ek8819@gmail.com (K.N.A.); amirayh@mans.edu.eg (A.Y.H.)

**Keywords:** big healthcare data, classification, decision-making, feature selection, whale optimization, naive bayes

## Abstract

There is a crucial need to process patient’s data immediately to make a sound decision rapidly; this data has a very large size and excessive features. Recently, many cloud-based IoT healthcare systems are proposed in the literature. However, there are still several challenges associated with the processing time and overall system efficiency concerning big healthcare data. This paper introduces a novel approach for processing healthcare data and predicts useful information with the support of the use of minimum computational cost. The main objective is to accept several types of data and improve accuracy and reduce the processing time. The proposed approach uses a hybrid algorithm which will consist of two phases. The first phase aims to minimize the number of features for big data by using the Whale Optimization Algorithm as a feature selection technique. After that, the second phase performs real-time data classification by using Naïve Bayes Classifier. The proposed approach is based on fog Computing for better business agility, better security, deeper insights with privacy, and reduced operation cost. The experimental results demonstrate that the proposed approach can reduce the number of datasets features, improve the accuracy and reduce the processing time. Accuracy enhanced by average rate: 3.6% (3.34 for Diabetes, 2.94 for Heart disease, 3.77 for Heart attack prediction, and 4.15 for Sonar). Besides, it enhances the processing speed by reducing the processing time by an average rate: 8.7% (28.96 for Diabetes, 1.07 for Heart disease, 3.31 for Heart attack prediction, and 1.4 for Sonar).

## 1. Introduction

**Figure 1:**
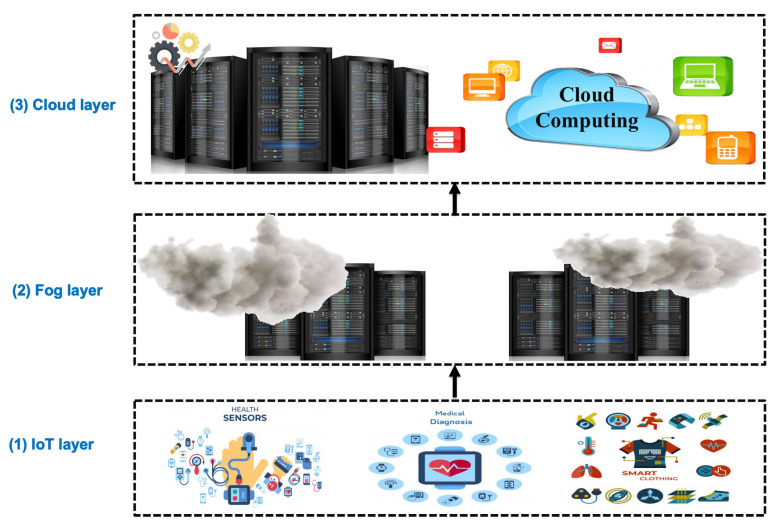
Modern healthcare systems’ structure.

Recently, many medical devices are equipped with sensors to collect, communicate, and integrate the massive generated medical data. Modern healthcare systems are based on emerging technology such as Wireless Sensor Networks (WSN) and the Internet of Things (IoT). Moreover, there is a widespread deployment for smart mobility initiatives that increase the development of intelligent healthcare systems. The objective is to maximize the use of real-time data streaming out of various medical, sensory services. The IoT generates diverse and complex big healthcare data. This data poses many challenges to the storage and analysis infrastructure. The convergence of IoT and several fundamental technologies such as cloud computing has become necessary to address the aforementioned challenges [1]. As shown in Figure 1, IoT-based healthcare systems may deploy a wide range of computing technologies such as cloud, edge, and fog computing, as a virtual resource utilization infrastructure.

**Figure 2:**
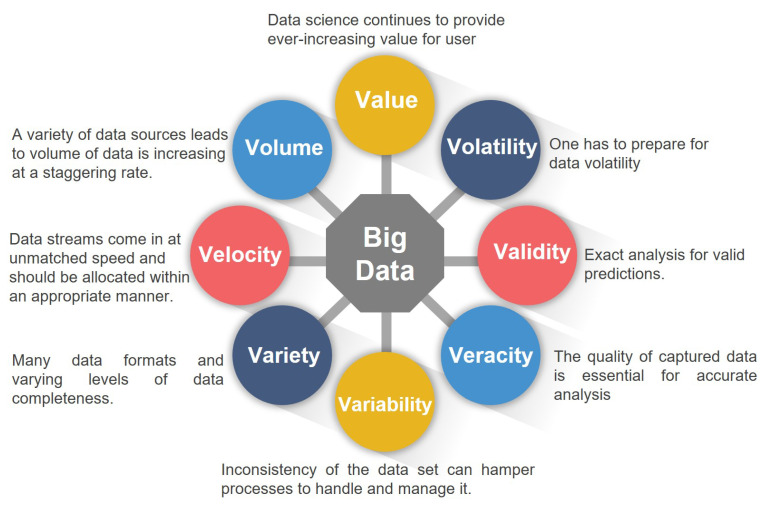
Big data multi-V’s model.

Big data has become a slogan for many scientific and technological enterprises, researchers, data analysts, and technical practitioners. Big data can be defined as any large and complex data source (gold mine) combined with a combination of old and new data-management technologies and architecture. Organizations can gather, store, manage, and manipulate extremely large volumes and a wide variety of data from many sources at the required speed and the proper time to gain the right insights [2]. Big data offers the basic functionalities that enable different organizations to manage data rapidly, timely conducted, and obtain smart decisions to gain the value of big data [3]. Big data is characterized by three V’s (Volume, Velocity, and Variety), according to industrial data analyst Doug Laney [4]. Three V’s are increased by four more V’s (Variability, Veracity, Validity, and Volatility) up to seven V’s later, as shown in Figure 2. To cope, the big biomedical data is characterized by scale, diversity, and complexity. Biomedical data processing consists of phases that are collecting, processing, and managing data. The main objective is to produce new information for end-users [5]. There are four steps for big data analysis, defined as four A’s: Acquisition, Assembly, Analyze, and Action.

The main objective of big data architecture is to extract value from a wide range of data by collecting the raw generated data from various data sources (Acquisition) [2]. Data collection techniques are used to collect raw data from various data formats. Analyze means using analytical methods, algorithms, and tools to find new insights and extract value. Data mining simultaneously helps to generate insight and forecasting patterns and provides smart query functions, then decisions (Action) must be available [6].

The biomedical domain also joins the era of the development of big data. The big data contains patient information, essential signals, and others from a wide range of data sources. Big data technology stores, analyzes, and exploits patient information. However, a cloud-based IoT healthcare system suffers from challenging problems that are demanding prompt solutions. The following list surveys some barriers [7] such as:

- The massive collected data storage;
- Eliminate privacy and security leakage at a different platform level;
- Energy management with continuous monitoring leads to an increase in data volume and analytical demands;
- Deliver the information at the proper time and in a reliable manner;
- Heterogeneity: the diversity of the connected things;
- High dynamics: the dynamic global network infrastructure;
- Quality of Service (QoS) supports both QoS and functional properties concerning a Service-Level Agreement (SLA).

Speed, efficiency, and high computational cost problems can be solved by saving time and reducing processing costs. We need to reduce the volume of data, and this can be implemented by reducing the feature of big data being processed. Data volume minimization can be achieved via the implementation of a Feature Selection (FS) technique. FS affects performance and offers faster decisions. FS determines the features that should be employed to improve performance [2].

The metaheuristic algorithms find the optimal settings of the application parameters and hyperparameters [8]. Metaheuristic algorithms can be categorized into three categories: evolutionary algorithms (EAs), trajectory-based algorithms, and swarm-based algorithms. Swarm-based algorithms are intuitive and inspired by nature, humans, and animals. While working with these algorithms, the researcher should make a compromise between exploration and exploitation. It turns out that the exploration process is searching far from the current candidate solution, while the exploitation is searching in the vicinity, near the current solution. The Whale Optimization Algorithm (WOA) has a low number of adjustable hyperparameters. The WOA mimics the humpback whale in searching for prey. The WOA consists of three operators to model the behavior of humpback whales. The WOA can accomplish data optimization missions by minimizing the number of features with high performance and making data ready to classify. The data are currently ready to be categorized, and several classification algorithms are included, including Decision Tree, Deep Learning (DL), K-Nearest Neighbor (KNN), and Naïve Bayes (NB). The NB classifier is a Bayes theorem-based model of probabilistic machine learning. NB can accomplish data classification as fast, simple to enforce, and real-time action support.

The main objective of this study is to propose a suitable approach for processing medical data rapidly in real-time and increasing its accuracy in a form that saves computational costs. This can be achieved by proposing an Ambient Healthcare approach with the Hybrid Whale Optimization Algorithm and Naïve Bayes Classifier (AHCA-WOANB) to perform feature selection on data and then classify it to reduce processing time while increasing performance.

The remaining of this paper will be as following: Section 2 will discuss related work and put a spotlight on the pros and cons of every discussed contribution; Section 3 will introduce the proposed AHCA-WOANB approach and the way of embedding the hybrid algorithm; Section 4 will introduce the experimental results that obtained; Finally, Section 5 will introduce the conclusion and future work.

## 2. Related Work

Medical services expect significant advancements through IoT and cloud computing integration. This integration introduces new forms of intelligent medical equipment and applications. The recently developed and introduced medical systems are targeted at the industry and academia to implement modern healthcare systems. IoT-based health architecture captures, processes, and analyzes medical data. In this vein, developing healthcare architectures, feature selection, and data classification has received significant attention in academia and the industry in the last few years [9]. In the next subsections, there will be a detailed description of the recent healthcare architecture. The WoA and NB classifier will also be surveyed.

### 2.1. Healthcare Architectures

Abawajy et al. [6] suggested a Cloud-based Patient monitoring architecture. There are three stages to their proposed architecture: collection station, data center, and monitoring station. Andriopoulou et al. [10] proposed a healthcare service framework based on fog computing that intermediates between clouds and loT devices and allows for new forms of computing and services. Their architecture comprises three main layers: data aggregation fog nodes, information storage, data processing and analysis fog servers, and data storage clouds. The same study introduced an IoT-based architecture for fog-based healthcare networks [10]. The design and implementation of the proposed architecture were in three layers. The first layer is IoT-based devices. The second layer consists of fog, while the third layer consists of the cloud layer. This architecture reduces cloud service traffic and provides low delays and immense permanent storage space. The integrated edge, fog, healthcare IoT-based cloud infrastructure was implemented by Dimosthenis et al. [11]. Their architecture consists of three layers for acquiring operation, data storage, and decision-making in real-time. The three layers are the edge layer that is close to the patients, the fog layer responsible for storing and processing data, and the cloud infrastructure that stores and analyzes data extracted from the fog and edge layers. Hassan et al. [9] have developed a 4-layer hybrid architecture named HAAL-NBFA, inspired by a growing interest in the use of AmI to develop care assistance systems for elderly patients. The HAAL-NBFA used both local monitoring and cloud-based architectures. The goal was to predict a patient’s health status from contextual circumstances. They suggested a five-stage cloud classification model that can deal with broad imbalanced datasets. The Deep Learning Three-Layer Architecture called HealthFog was proposed by Shreshth Tuli et al. [12]. HealthFog shows its performance in energy usage, latency, and execution time. QoS attributes are not taken into account. The comparison of recent health system architectures in the literature is shown in Table 1.

### 2.2. Whale Optimization Algorithm

One of the well-known metaheuristic optimization algorithms is the Whale Optimization Algorithm (WOA) [13,14]. WOA is considered a Wrapper-based Feature Selection technique, influenced by nature, proposed by Seyedali Mirjalili et al. [13,14]. The main inspiration for WOA is the actions of humpback whales. Whether by encircling or bubble-net approaches, they strike the prey. The current optimal location in the surrounding activity is treated as the prey, and according to Equations (1) and (2), the whale updates its position.

(1) $$\vec{D}=\left|C.\vec{X^{*}}\left(t\right))-X\left(t\right)\right|$$

(2) $$\vec{X}\left(t+1\right)=\vec{X^{*}}\left(t\right)-\vec{A}.\vec{D}$$

where *t* refers to the current iteration, $X^{*}$ is the vector that corresponds to the best solution, and *X* defines the position vector of the whale. The absolute value is || and . is the element-wise multiplication. $\vec{A}$ and $\vec{C}$ are determined as follows in Equations (3) and (4).

(3) $$\vec{A}=2\vec{a}.\vec{r}-\vec{a}$$

(4) $$\vec{C}=2.\vec{r}$$

where *a* is linearly decreased from 2 to 0 throughout iterations, and *r* indicates a random number in [0,1].

**Figure 3:**
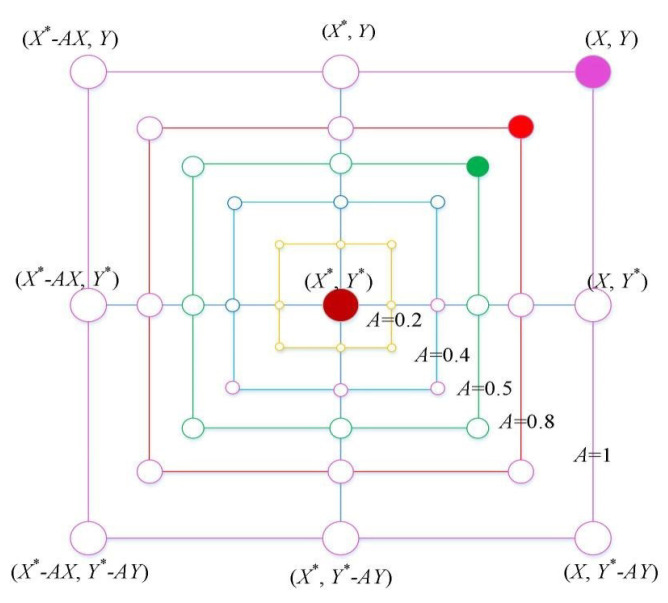
The WOA shrinking encircling mechanism.

There are only two ways to simulate bubble-net behavior. The first is to shrink the enclosing using Equation (3) with a reduced range of *A* by *a*. The search agent’s new position can be defined anywhere between the best possible current position and the original position. Figure 3 depicts the feasible position from (X,Y) to $\left(X^{*},Y^{*}\right)$ that $\vec{A}$ can obtain in a 2D space, as given by Equation (3). The second one is the spiral updating positions; Equation (5) is used as a logarithmic spiral equation. The movement of humpback whales around the prey is helix-shaped, which is mimicked using Equation (5).

(5) $$\vec{X}\left(t+1\right)=\vec{D^{{}^{\prime }}}.e^{bl}.cos\left(2\pi l\right)+\vec{X^{*}}\left(t\right)$$

**Figure 4:**
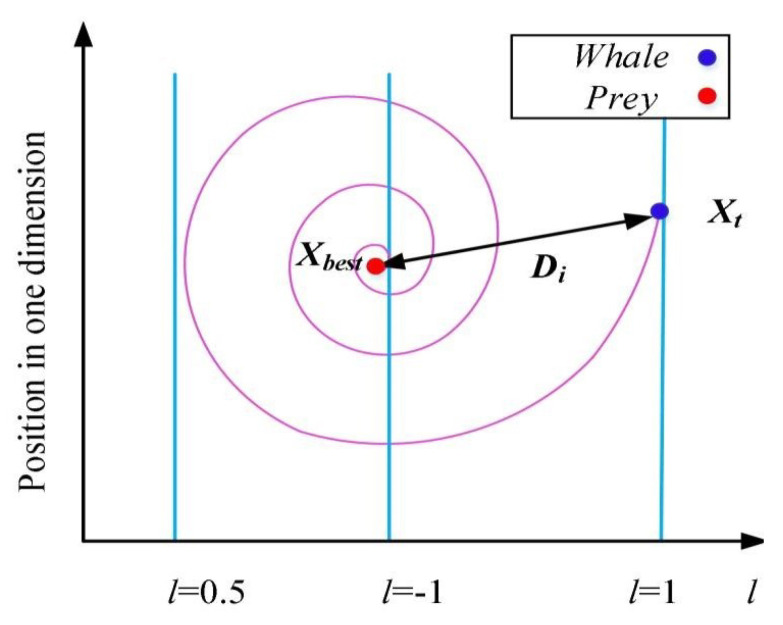
The spiral updating position.

Here, $\vec{D^{{}^{\prime }}}=\left|\vec{X^{*}}\left(t\right)-X\left(t\right)\right|$ is the distance from the *i*th whale to the victim, and *B* is a parameter for determining the form of the logarithmic spiral. *l* denotes a random number in [−1,1] that determines how close the next location of the whale is to the victim. l=−1 is the nearest location to the victim as shown in Figure 4.

It is worth remembering that humpback whales will simultaneously swim around the prey and along spiral-shaped tracks in a shrinking circle. To model this concurrent activity, the researchers believe that the processes of shrinking or the spiral model for adjusting the whale’s location are equally probable. Equation (6) defines the mathematical model as follows.

(6) $$\vec{x}\left(t+1\right)=\left\{\begin{array}{cc} \vec{X^{*}}\left(t\right)-\vec{A}.\vec{D} & \;\mathrm{if}\;p<0.5\\ X\left(t+1\right)=\vec{D^{{}^{\prime }}}.e^{bl}.cos\left(2\pi l\right)+\vec{X^{*}}\left(t\right)\; & \;\mathrm{if}\;p\geq 0.5 \end{array}\right.$$

Here, *p* is a random number in [0,1], which decides when to use the spiral model or the shrinking encircling method to change the whale position. In addition, humpback whales will search randomly, depending on the location of each other. The mechanism can be accomplished as follows:

(7) $$\vec{D}=\left|\vec{C}.X_{rand}^{\to }\left(t\right)-\vec{x}\left(t\right)\right|$$

(8) $$\vec{X}\left(t+1\right)=X_{rand}^{\to }\left(t\right)-\vec{A}.\vec{D}$$

where $X_{rand}^{\to }$ is a random whale (a random position vector) chosen from the current population. The WOA algorithm’s pseudo-code is shown in Algorithm 1. The WOA algorithm randomly chooses *X* as the optimal way to enhance exploration.

The $X^{*}$ value is chosen in the WOA algorithm for moving randomly selected whales rather than the best one to boost exploration. Besides Features Selection as a way to process data, there are other methods, including data classification. Data classification can be done in more than one form and by using many algorithms that differ in how they classify the big data.
**Algorithm 1:** The WOA
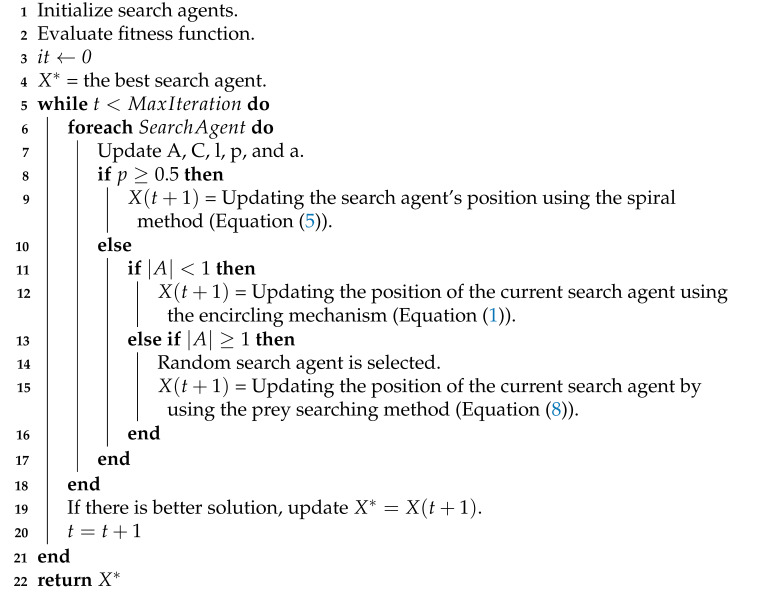


### 2.3. Naïve Bayes Algorithm

The Naïve Bayes Algorithm (NB) is a Bayes Theorem-based classification technique with an assumption of independence among predictors. It can be used for spam filters, text analysis, and medical diagnosis [15]. Naïve Bayes is considered one of the best algorithms with several advantages, such as easy implementation, high speed, and efficiency. NB requires less training data, is scalable, handles both continuous and discrete data, and is best suited for text data and fog computing support. The Naïve Bayes model is simple to construct and especially effective for very large datasets. Naïve Bayes also provides highly advanced classification methods as well as simplicity. The theorem of Bayes provides a way of calculating posterior probability P(c∣x) from P(c), P(x), and P(x∣c). The equation will be:

(9) $$P\left(c|x\right)=\frac{P(x|c).P(c)}{P(x)}$$

The equation parameters are:

- P(c∣x): the posterior probability of class (*c*, target) given predictor (*x*, attributes).
- P(c): the prior probability of class.
- P(x∣c): the likelihood which is the probability of the predictor given class.
- P(x): the prior probability of the predictor.

The classification process can easily be described in three simple steps: (i) create the frequency table from the dataset, (ii) establish a Likelihood table by specifying the probabilities, and (iii) use the Bayesian equation to measure the post-class probability. The prediction result is the class with the highest posterior probability. In practice, it is nearly impossible to obtain a set of completely independent predictors. Assume the categorical variable in the test data has a category but not in the train data; in this case, the probability of this category is set to zero, and prediction is impossible.

To summarize, medical data has a very large size and has many features that can be decreased to make processing faster. There is a need to find a suitable method for processing medical data rapidly in real-time and increasing its accuracy in a form that saves computational cost. Many attempts were spotted on this point, and many solutions were introduced but with drawbacks in processing time and performance.

## 3. Methods

### 3.1. The Ambient Intelligent Healthcare Approach

Data evolves over time in most challenging data analysis applications and must be analyzed in near real-time. Patterns and relationships in such data frequently evolve over time, so models built to analyze such data quickly become obsolete. This phenomenon is known as concept drift in machine learning and data mining. In machine learning and data mining, concept drift refers to changes in the relationships between input and output data in the underlying problem over time. There are several approaches to dealing with concept drift; the most common is ignoring it and assuming that the data does not change. If you suspect that your dataset may be subject to concept drift, you can use a static model to detect Concept Drift Detection and a Baseline Performance. This should be your starting point and benchmark for comparing other methods. Solving the problem of increased processing time and high computational cost for medical big data systems is crucial. This can be achieved via (i) proposing an approach for processing various types of medical data, (ii) predicting useful information with minimum computational costs, and (iii) processing data in real-time. Therefore, a hybrid algorithm that consists of two phases is proposed. First, a feature selection technique is used to minimize the number of features. Thereafter, the second phase of the proposed hybridized algorithm is data classification.

**Figure 5:**
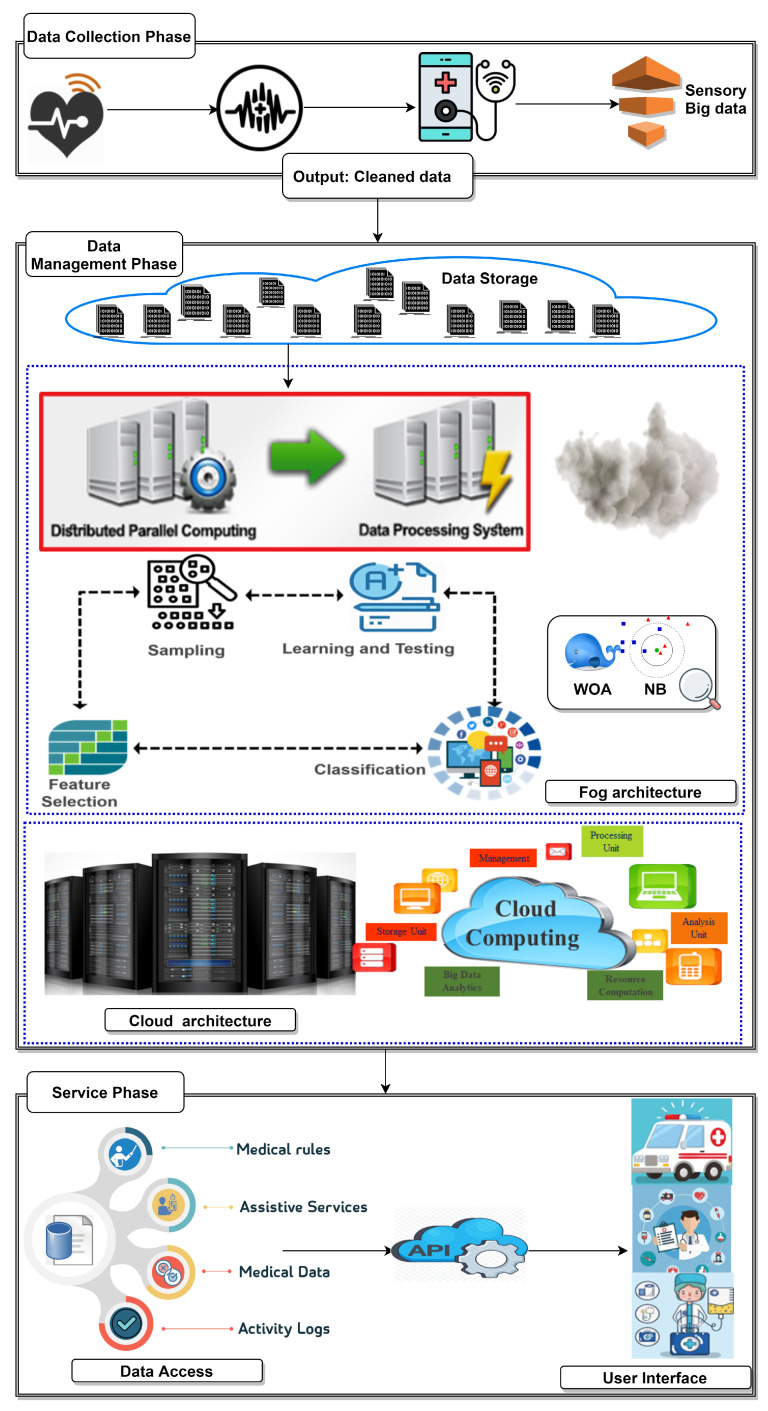
The proposed Ambient Intelligent Healthcare approach.

As shown in Figure 5, the block structure of the proposed Ambient Healthcare approach with the Hybrid Whale Optimization Algorithm and the Naïve Bayes Classifier (AHCA-WOANB) consists of three main phases, which are the data collection phase, data processing phase, and services layer. Based on fog computing, the AHCA-WOANB gains most of its benefits, including enhanced business agility, improved security, deeper privacy knowledge, and reduced cost of operation.

The proposed approach phases are working according to specific steps. The first phase starts collecting data from various sources. Data diversity is concerned at this phase. For performing the data management process, data are transferred to the second phase. In the second phase, data are stored, then optimized and classified in a suitable way that facilitates the third phase to work correctly and introduce perfect services. In the next sections, there will be a detailed description of the phases of the AHCA-WOANB approach.

#### 3.1.1. The Data Collection Phase

This phase consists of two steps: one for data perceptions and the second one responsible for transferring collected data to the next phase. The data comes from various sources such as hospitals, research institutes, wearable devices, and public organizations. After that, the collected data is transferred to the next phase via a networking medium.

#### 3.1.2. The Data Management Phase

Fog technology is used to provide low latency and real-time communication between the data management phase and the other phases. To this end, this phase is applied using Hadoop [16,17], which is an open-source, Java-based software framework. The main objective of deploying Hadoop is to distribute data stores and applications processing on large clusters.

Hadoop provides massive storage for any kind of data, which is called the Hadoop Distributed File System (HDFS), and enormous processing power that is accomplished by Hadoop MapReduce programming, and this processing is easily made based on parallel computing. These support Hadoop with the ability to handle virtually limitless concurrent tasks or jobs and make it highly fault-tolerant and deployable on low-cost hardware. All of this makes it easy to depend on Hadoop as a backbone of any modern big data framework.

The data management phase consists of two modules that are responsible for data storage and processing. The first module is data storage, in which data are stored in the HDFS [16]. HDFS can store and spread massive datasets on hundreds of low-cost parallel servers. This supports the proposed approach with cost efficiency, flexibility, speed, and resilience to failure. The second module is data processing and classification, which uses Hadoop MapReduce [17] programming based on the proposed hybrid algorithm (WOA for feature selection then NB for classifying) and parallel computing to process many types of data.

**Figure 6:**
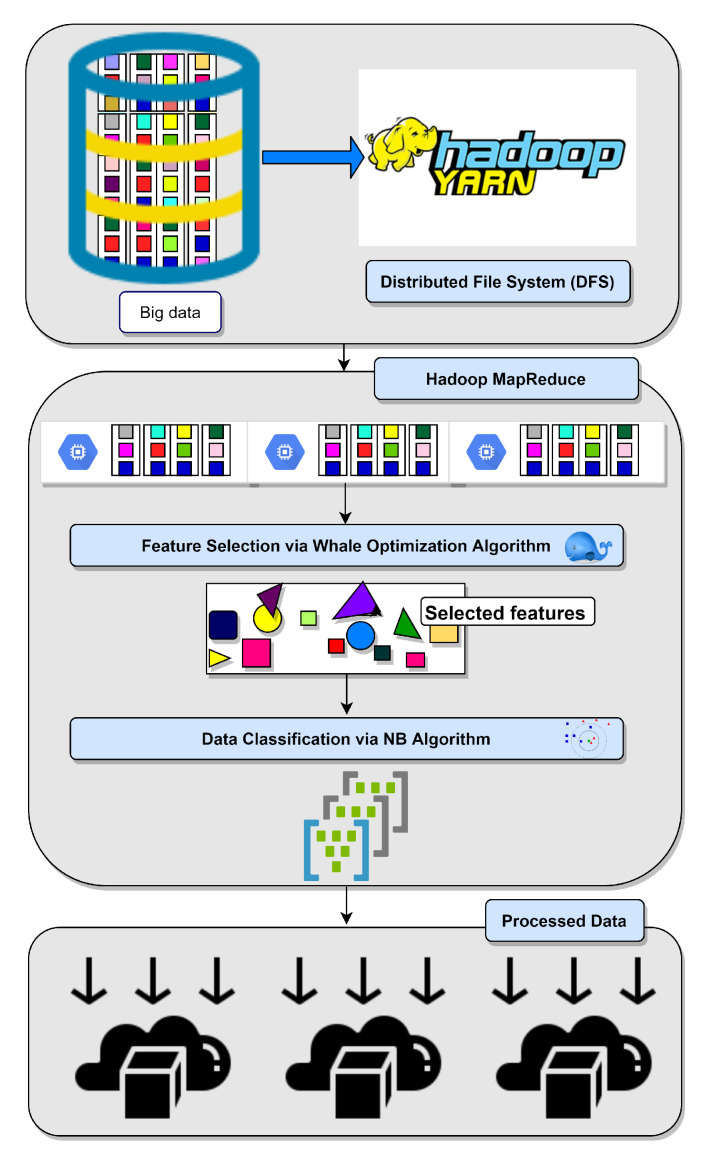
The proposed AHCA-WOANB approach data processing steps.

The processing in this phase means optimizing data by using a hybrid algorithm. This algorithm performs a feature selection on big data that is stored in the HDFS using WOA, then classifies this optimized data using NB, and this processing is accomplished by MapReduce programming and parallel computing, as shown in Figure 6.

**Figure 7:**
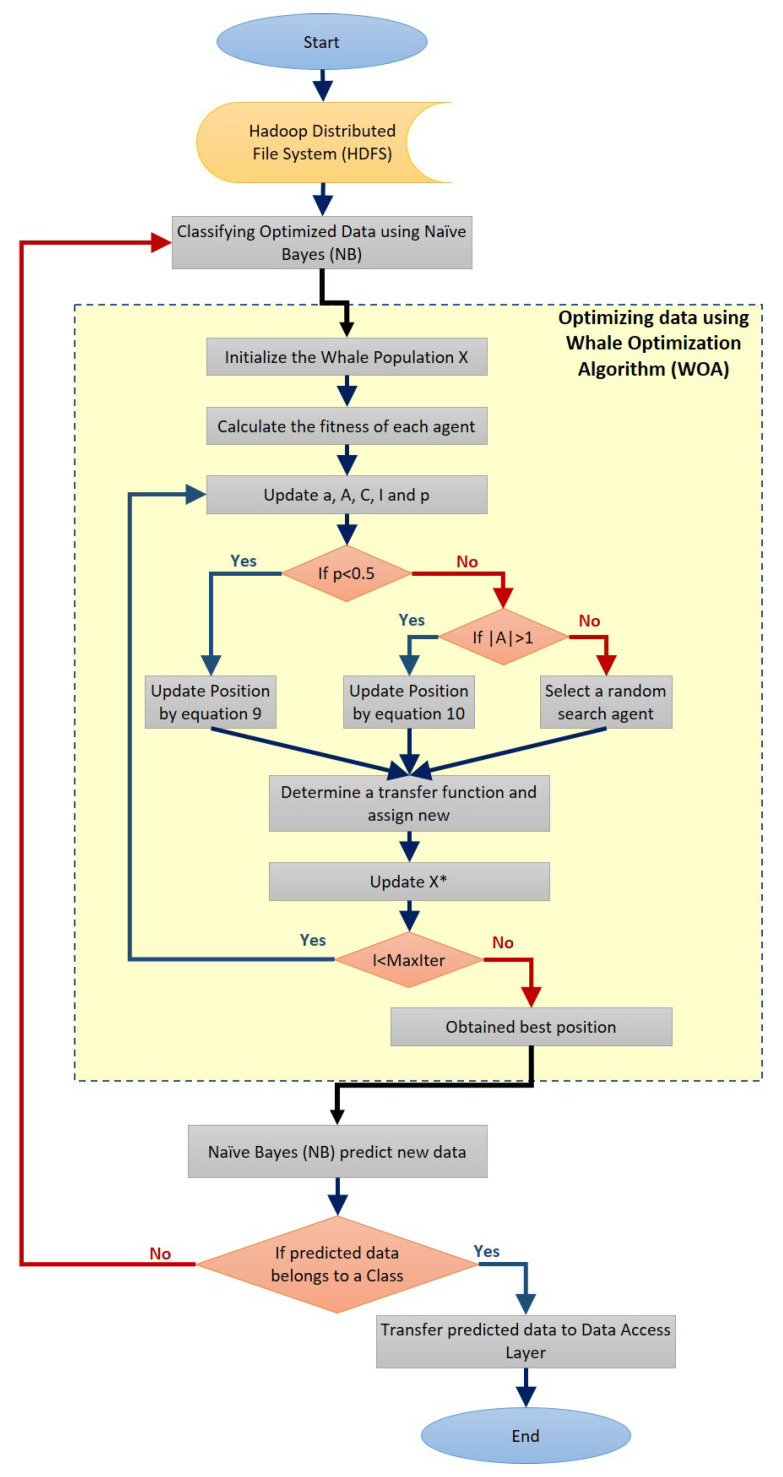
The proposed AHCA-WOANB approach flowchart.

Data optimization and classification, as shown in Figure 7, are performed using MapReduce programming and parallel computing. This step is executed with the WOA for optimizing data by reducing the number of features of the currently processed dataset.

Whales in the classical WOA move within the continuous search space to change their positions, referred to as continuous space. However, to solve Feature Selection problems, the solutions are limited to only 0 and 1 values. Therefore, continuous (free position) solutions must be converted to binary solutions to solve feature selection problems. As a result, a binary version of WOA is introduced to investigate the Feature Selection problem. The conversion is carried out by utilizing specific transfer functions, such as the S-shaped function. As a result, several studies have considered that the FS problem is an optimization problem; thus, the fitness function for the optimization algorithm has been changed to classifier accuracy, which the chosen features may maximize.

In this case, the proposed WOA algorithm is used to find the best features in an adaptive feature space search. This combination is obtained by achieving the highest classification accuracy while using the fewest features. The fitness function is depicted in Equation (10) below and the two proposed versions for evaluating individual whale positions.

(10) $$F=\alpha \gamma_{R}\left(D\right)+\beta \frac{\left|C-R\right|}{\left|C\right|}$$

where:

- *F* denotes fitness function.
- *R*: the length of the selected feature subset.
- *C*: the total feature numbers.
- $\gamma_{R}\left(D\right)$: classification accuracy of the subset with length *R*.
- α: argument ∈[0,1].
- β: argument =1−α.

As a result, the fitness function with the highest classification accuracy will be produced. Based on the classification error rate and selected features, the equation above can be converted to a minimization problem. As a result, the obtained minimization problem can be solved, as shown in Equation (11).

(11) $$F=\alpha E_{R}\left(D\right)+\beta \frac{\left|R\right|}{\left|C\right|}$$

where $E_{R}\left(D\right)$ is the classification error.

The method entails dividing a dataset into two subsets. The first subset, referred to as the training dataset 70%, is used to fit the model. The second subset is not used to train the model; rather, the model is fed the dataset’s input element, and predictions are made based on the expected values. The second dataset is referred to as the test dataset 30%. The NB algorithm received the optimized datasets and started its mission to classify them and prepare for the predicting data stage. This means that while fewer features result in less computational complexity (both storage and execution), fewer features usually result in less accurate results due to the absence of useful information. The exception to this is when there are outliers and irrelevant features.

#### 3.1.3. The Service Phase

The service phase consists of a set of modules: data access, Application Programming Interface (API), and User Interface (UI) modules. These modules interact with each other for performing the appropriate decision making. The data access module receives data and statistics from the processing and classification module then prepares the data to be used with the API and UI modules.

## 4. Simulation and Computer Results

This section evaluates the performance of the proposed AHCA-WOANB approach. The performance metrics that the system is seeking to improve are:

1. Accuracy: The validity of the predicted data by the system; improving this factor makes the decision making easier and more convenient.
2. Time: The time that the system will take to classify the data; eliminating this factor will minimize the cost.
3. Data Variety: The amount of accepted data by the system; this indicates how flexible the approach is by accepting more forms of data.

### 4.1. Used Datasets and Physical Meaning

This section explores the common datasets that were obtained from Kaggle [18]. These datasets will be used to test the approach and produce results. They are also various types, and the proposed approach will accept them easily, as mentioned in the first phase’s description. Table 2 summarizes the characteristics of the used datasets.

#### 4.1.1. Diabetes

The dataset comes from the Diabetes and Digestive and Kidney Diseases National Institute. The dataset’s purpose is to predict based on certain measures contained in the dataset whether a patient has diabetes or not. The collection of these instances from a large database has been limited by many constraints. All patients here are women of Pima’s Indigenous Heritage who are at least 21 years old. The dataset contains multiple variables of the medical indicator and one variable objective, Outcome. Predictor variables (e.g., the number, BMI level, insulin level, age, and so on) of pregnancies that the patient has had.

#### 4.1.2. Heart Disease Uci

There are 76 attributes in this database, but recent research refers to the use of a subset of 14. The only one used by ML researchers to date was the Cleveland database. The target area applies to the patient’s involvement in heart disease. The integer value is between 0 (no presence) and 4. This set of data includes age, sex, type of chest pain (4 values), blood pressure, serum cholesterol in mg/dL, fasting blood sugar >120 mg/dL, electrocardiographic rest results (values, 1.2), achieved maximum heart rate, exercise inducing angina, exercise-induced ancient peak = ST exercise-induced depression, peak ST slopes, and the number of major vessels (0–3).

#### 4.1.3. Heart Attack Prediction

The content of the cardiac disease directory is listed in this database. The data collection consists of various sources, including the Cleveland Clinical Foundation (Cleveland data) and the University Hospital, Zurich, Switzerland (SWID). Data are available from a wide variety of sources, including the VA Medical Center, Long Beach, CA (long-beach-va.data).

#### 4.1.4. Sonar

This data collection includes 60 patterns derived from photos during pregnancy, which are used to assess fetal biometrics through ultrasound imagery. One such measurement is the circumference of the fetal head (HC). The HC can be used to estimate the pregnancy and to track fetal development. In a certain cross-section of the fetal head, called the default plane, HC is calculated. A total of 1334 2D images of the Standard Plane can be used to calculate the HC in the dataset for this challenge. In this challenge, algorithms built to calculate the fetal head circumference automatically can be compared in 2D ultrasound images.

### 4.2. Computer Results

**Figure 8:**
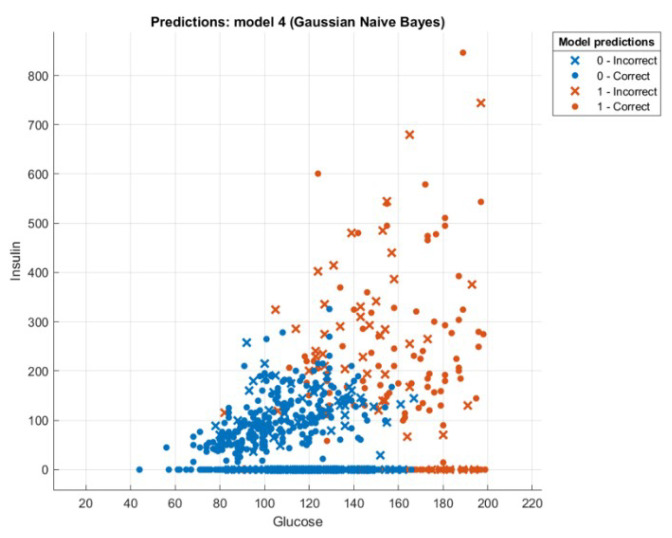
The Diabetes original and predicted data.

**Figure 9:**
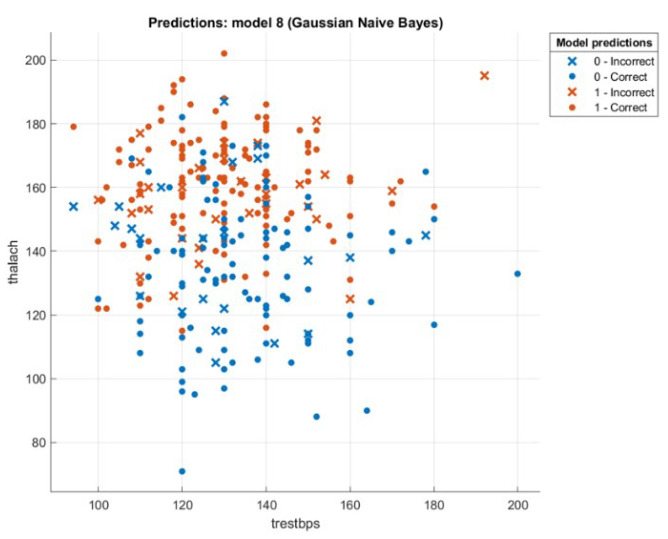
The Heart-C original and predicted data.

**Figure 10:**
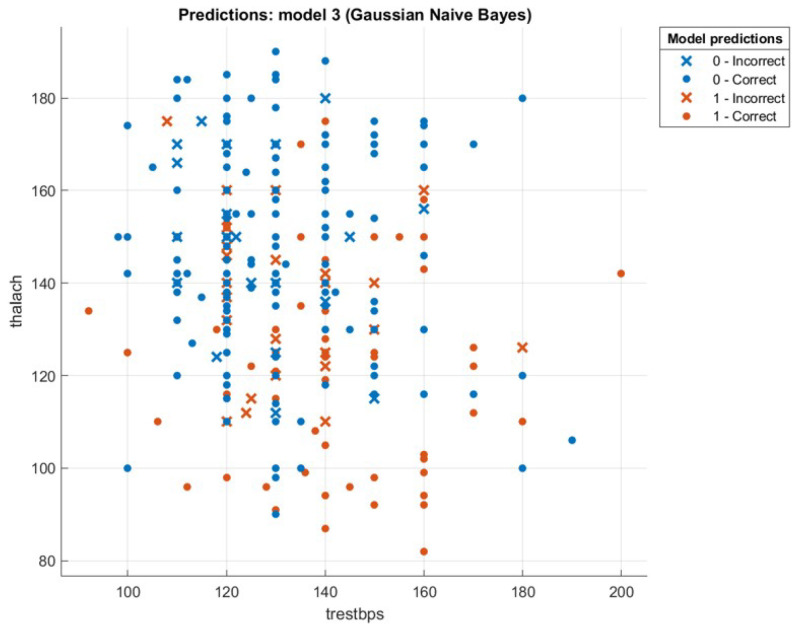
The Heart-H original and predicted data.

**Figure 11:**
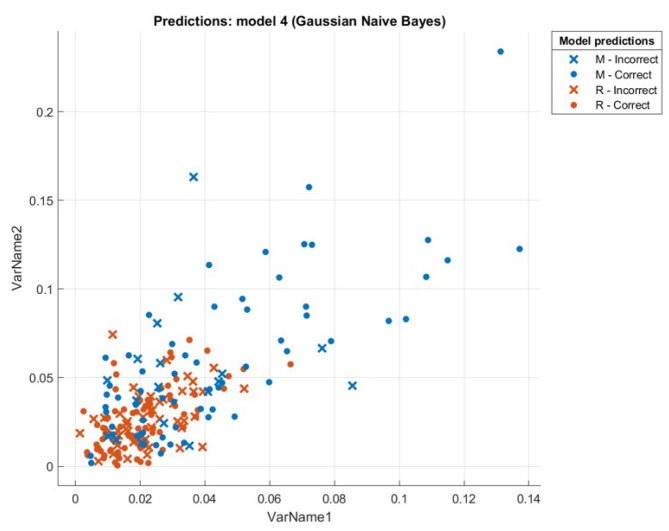
The Sonar original and predicted data.

This section presents the results that were achieved from testing the hybrid WOA-NB algorithm. First, we will introduce a comparison between the accuracy and speed of processing for every tested dataset using two ways. The first one is by executing classification only by using NB. The second one is by executing feature selection then classification by using WOA then NB, and this is the hybrid algorithm mentioned previously. The first-way results are introduced in Liangxiao et al. [19], which give NB results without other algorithms on multiple datasets. The second-way results will be calculated after executing the proposed hybrid algorithm. The comparison results will be shown in Table 3. Figure 8, Figure 9, Figure 10 and Figure 11 depict the original and predicted data shapes for different datasets.

**Figure 12:**
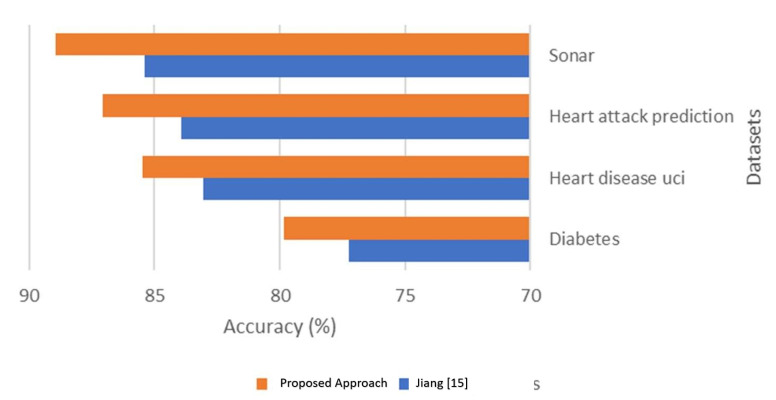
Accuracy comparison between NB and the proposed approach.

**Figure 13:**
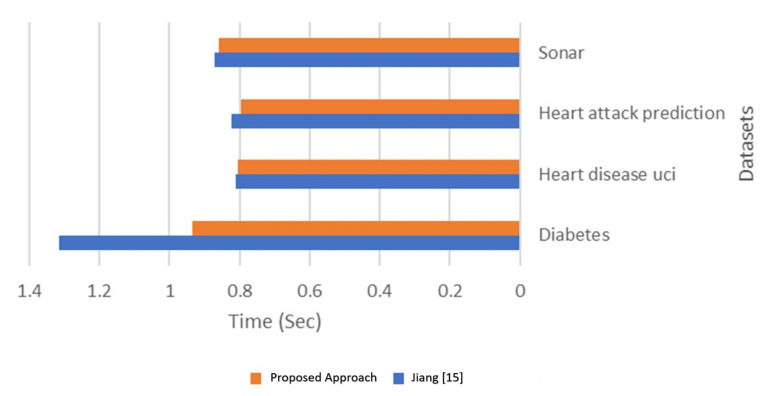
Time comparison between and the proposed approach.

The results show an enhancement in accuracy and time using the proposed approach over classification with only NB [19]. The enhancement is based on the number of reduced features after applying the WOA feature selection technique. In the Diabetes dataset, there are four of eight fewer features, and this enhanced the accuracy by 3.34% and reduced the computational time by 28.96%. In the Heart disease UCI dataset, there are 12 of 13 reduced features, and this enhanced the accuracy by 2.94% and reduced computational time by 1.07%. In the Heart attack prediction dataset, there are 12 of 13 reduced features, and this enhanced the accuracy by 3.77% and reduced the computational time by 3.31%. There are 52 of 60 fewer features in the Sonar dataset, which enhanced the accuracy by 4.15% and reduced the computational time by 1.4%. Figure 12 and Figure 13 compare accuracy results and processing time results calculated from both Jiang [19] and the proposed approach.

**Figure 14:**
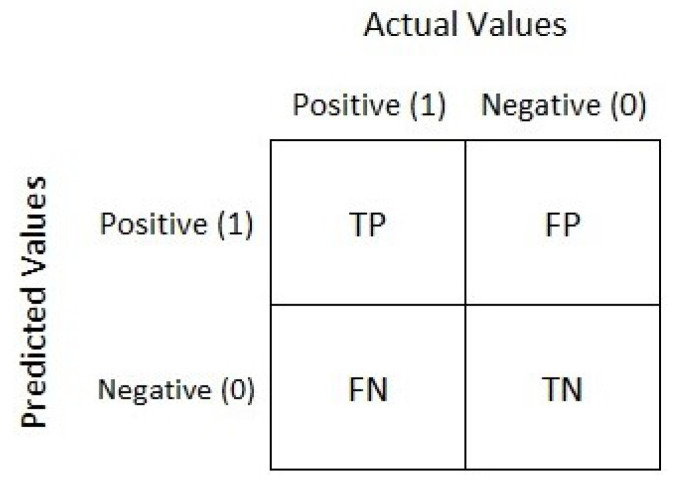
The confusion matrix.

The confusion matrix [20] is a performance calculation for a classification problem of learning machines that can measure the effectiveness of the proposed approach. The output can be two or more classes, as shown in Figure 14. It is a table of four different expected and true value combinations.

There are two classes (Class 1: Positive and Class 2: Negative), and there are many terms as follows: Positive (P), Negative (N), True Positive (TP), False Negative (FN), True Negative (TN), and False Positive (FP) as follows:

- Positive (P): Observation is positive (for example: is an apple).
- Negative (N): Observation is not positive (for example: is not an apple).
- True Positive (TP): Observation is positive and is predicted to be positive.
- False Negative (FN): Observation is positive but is predicted negative.
- True Negative (TN): Observation is negative and is predicted to be negative.
- False Positive (FP): Observation is negative but is predicted positive.

The Classification Rate or Accuracy can be calculated from Equation (12). Now, the confusion matrix results of the proposed WOA-NP algorithm are depicted in Table 4. Precision, as in Equation (13), tells us how many samples were actual positive out of all positive predicted samples. Recall, Equation (14), tells us how many positive samples were detected out of all actual positive samples.

(12) Accuracy=(TP+TN)/(TP+TN+FP+FN)

(13) Precision=(TP)/(TP+FP)

(14) Recall=(TP)/(TP+FN)

Sensitivity represents a positive data points proportion, which is correctly considered positive to all positive data points and calculated using Equation (15).

(15) $$Sensitivity=\frac{TP}{TP+FN}$$

Specificity is a negative data point proportion that is incorrectly considered positive to all negative data points. It can be calculated using Equation (16).

(16) $$Specificity=\frac{TN}{FP+TN}$$

The confusion matrix is useful to calculate the Recall, Precision, Specificity, and most significantly, the Receiver Operating Characteristic (ROC) curve (simply AUC) [21], and the confusion matrix is also useful for accuracy. The ROC curve is a graphical approach to demonstrate the difference between a classifier’s true-positive and false-positive rates. This allows for an approach under the ROC curve (AUC) to determine which classifier is on average better.

**Figure 15:**
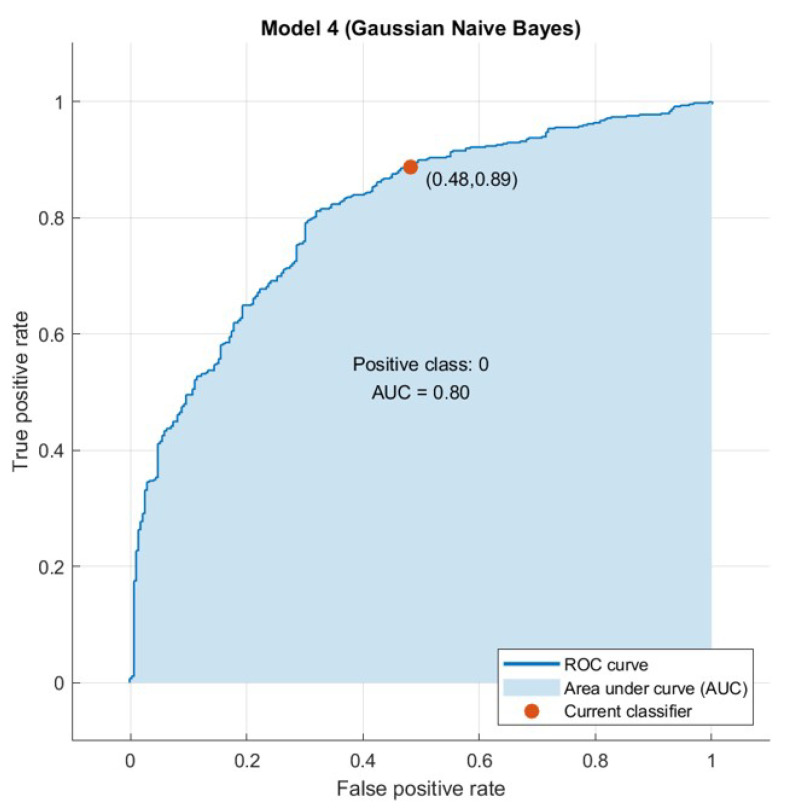
The ROC curve: Diabetes.

**Figure 16:**
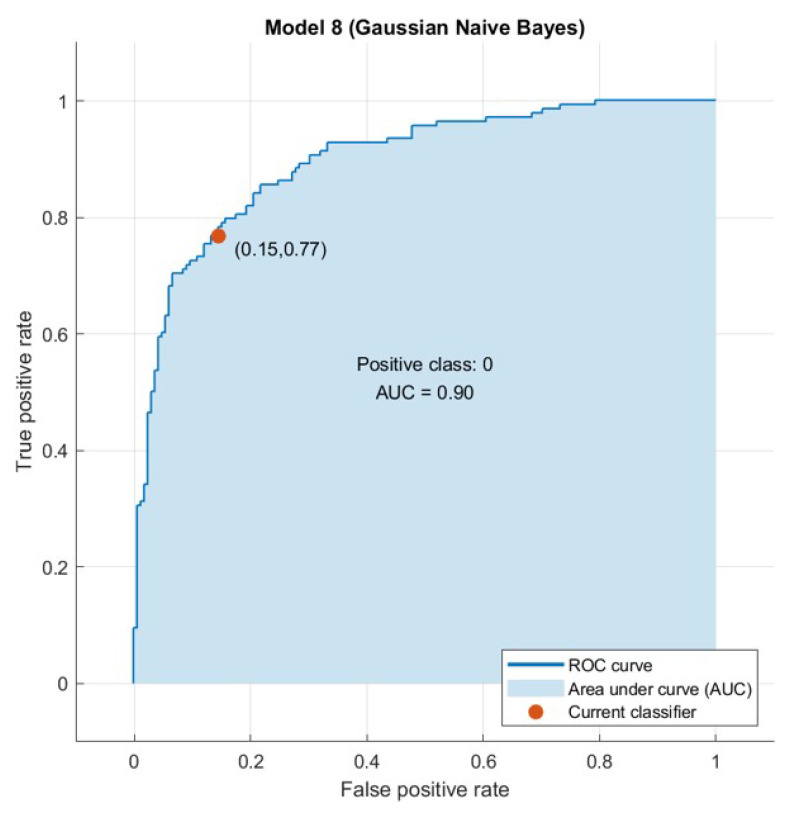
The ROC curve: Heart-C.

**Figure 17:**
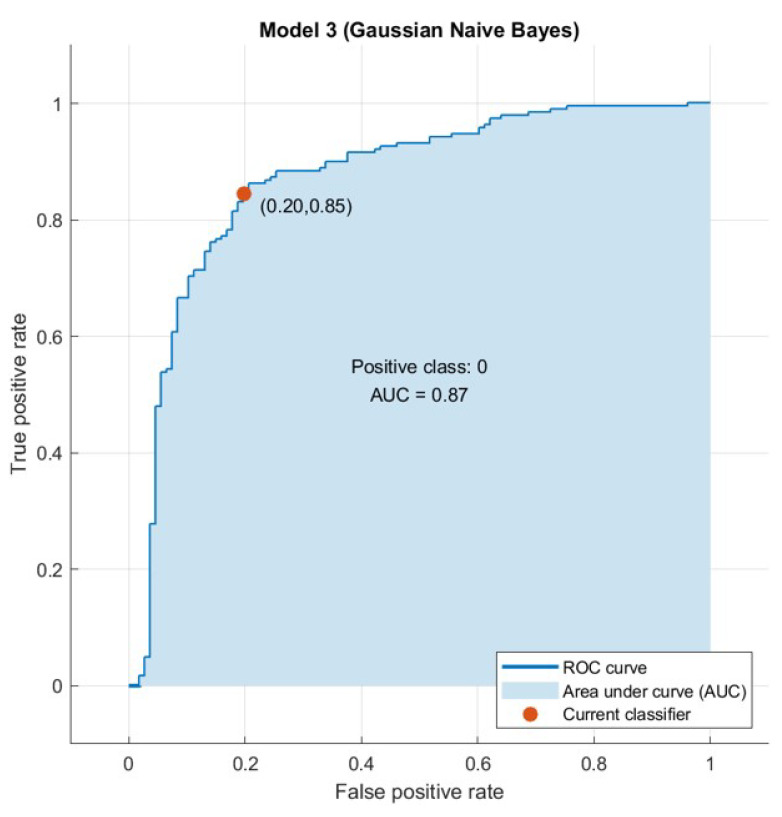
The ROC curve: Heart-H.

**Figure 18:**
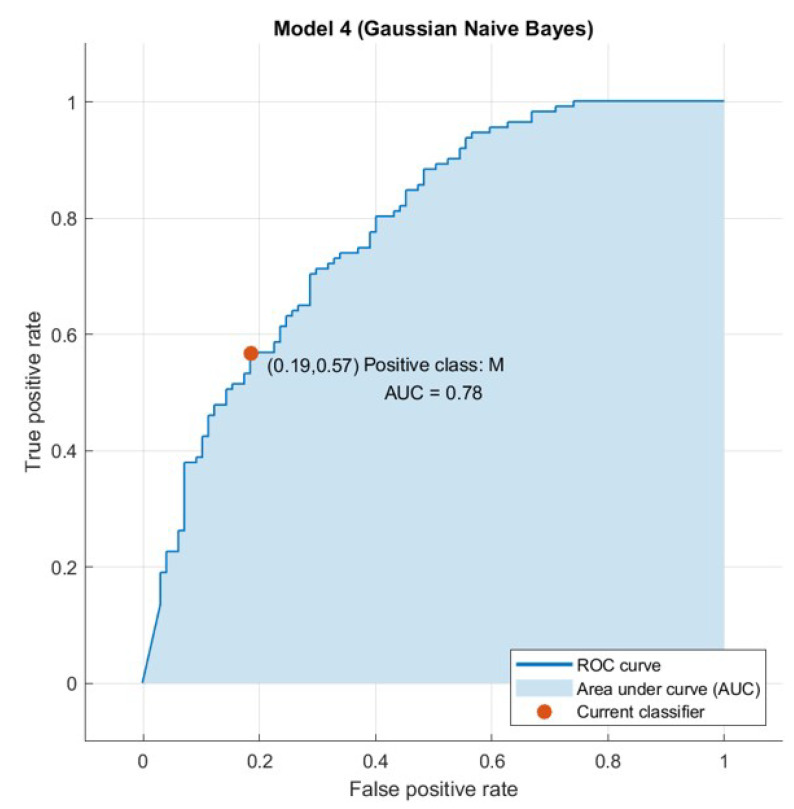
The ROC curve: Sonar.

AUC is a threshold invariant of classification. It tests the accuracy of model’s predictions regardless of the classification threshold selected. This implies the classifier is the greater the area under the curve more efficiently. Furthermore, there is a point on the curve that represents the optimal operating point of the classifier. Figure 15, Figure 16, Figure 17 and Figure 18 show the ROC curves for every tested dataset while processing. From these curves, we notice that the area under every curve is excellent, proving that the AHCA-WOANB approach classification is efficient.

Finally, all of these results lead us to clearly determine that the AHCA-WOANB hybrid algorithm (WOA for optimization and NB for classification) increases and enhances the accuracy by the average rate: 3.6% (3.34 for Diabetes, 2.94 for Heart disease UCI, 3.77 for Heart attack prediction, and 4.15 for Sonar) also can enhance the processing speed by reducing the processing time by the average rate: 8.7% (28.96 for Diabetes, 1.07 for Heart disease UCI, 3.31 for Heart attack prediction, and 1.4 for Sonar). The rate of these improved results, which are based on Datasets’ Characteristics, should be aware that whenever the optimization step can reduce the number of dataset features, this will improve the accuracy and reduce the processing time even more than improving them for those datasets that have less few features.

## 5. Conclusions

Many healthcare big data needs too much effort to give humanity useful information that can help develop and enhance this field reasonably with the low computational cost. Therefore, the AHCA approach with a hybrid algorithm has been proposed to process various types of medical data. Then it can be easy for us to predict data and introduce useful information and statics to submit it to several parties that concerned this area. The AHCA-WOANB approach has two steps of processing. This is to optimize data to make the second one more efficient, while the second one is responsible for classifying the optimized data. The proposed algorithm increases and enhances the accuracy by approximately 4%. It can also enhance the processing speed by reducing the processing time by approximately 9%. (These results are the average of the results for all tested datasets that are based on characteristics of data and the number of features that have been reduced by the WOA.)

The future mission is to try to support the proposed algorithm by modifying the WOA parameters set automatically by using a conventional neural network algorithm to get better results because it optimizes the used data perfectly before it is processed with the NB algorithm. This will reduce human interactions, so it will reduce human mistakes, reduce the duration time of processing, and give better accuracy than before.

## Figures and Tables

**Table 1:** Recent healthcare system architecture.

Architecture	No. of Layers	Scalability	Flexibility	Real-Time Support	Energy-Efficiency	Computational Cost
PPHM [6]	Three Layer	Scalable	Flexible	N/A	Energy-efficient	High
HSDA [10]	Three Layers	Moderate	Moderate	support	Moderate	Moderate
EFCHioT [11]	Three Layers	Scalable	Limited	support	Energy-efficient	High
HAAL-NBFA [9]	Four Layers	Scalable	Limited	support	Moderate	High
HealthFog [12]	Three Layers	Limited	Moderate	support	Energy-efficient	Low

**Table 2:** The characteristics of the used datasets.

Dataset	# Instances	# Features	Clasisfication Type	Availability
Heart disease UCI	303	14	Multiclass	The data set is publicly available on the Kaggle website https://www.kaggle.com/ronitf/heart-disease-uci (accessed on 2 July 2021)
Pima Indians Diabetes Database	768	9	Binary class	The data set is publicly available on the Kaggle website https://www.kaggle.com/uciml/pima-indians-diabetes-database (accessed on 2 July 2021)
Heart Attack Prediction	294	76	Multiclass	The data set is publicly available on the Kaggle website https://www.kaggle.com/imnikhilanand/heart-attack-prediction (accessed on 2 July 2021)
Sonar	1334	60	Binary class	The data set is publicly available on the Kaggle website https://www.kaggle.com/ypzhangsam/sonaralldata (accessed on 2 July 2021)

**Table 3:** Accuracy and speed comparison between NB and WOA-NB.

Classifier		Datasets			
**Algorithm(s)**	**Parameters**	**Diabetes**	**Heart-C**	**Heart-H**	**Sonar**
	No. of Features	8 of 8	13 of 13	13 of 13	60 of 60
NB	Accuracy (%)	77.24	83.04	83.91	85.4
	Time (s)	1.3151	0.81224	0.82374	0.87044
	No. of Features	4 of 8	12 of 13	12 of 13	52 of 60
WOA and NB	Accuracy (%)	79.82	85.48	87.07	88.94
	Time (s)	0.93421	0.80358	0.79651	0.85827

**Table 4:** The confusion matrix results.

Datasets/Metrics	TP	FP	FN	TN	Precision	Recall	Specificity	Sensitivity
Diabetes	4730	410	1140	1400	92%	80.57%	77%	81%
Heart disease uci	1120	260	180	1470	81%	86.15%	85%	86%
Heart attack prediction	1660	250	130	900	82%	90%	78%	93%
Sonar	980	130	100	870	88%	91%	87%	91%

## Data Availability

Data available upon request.
